# Functional quadriceps endurance, dynamic balance, and patient-reported recovery after total knee arthroplasty: a cross-sectional analysis of functional capacity and perceived disability

**DOI:** 10.3389/fmed.2025.1747975

**Published:** 2026-01-21

**Authors:** Saleh Kardm, Tariq Abdullah Aldugman, Ravi Shankar Reddy, Faisal M. Alyazedi

**Affiliations:** 1 Department of Surgery, College of Medicine, Najran University, Najran, Saudi Arabia; 2 Program of Physical Therapy, Department of Medical Rehabilitation Sciences, College of Applied Medical Sciences, King Khalid University, Abha, Saudi Arabia; 3 Department of Physical Therapy, Prince Sultan Military College of Health Sciences, Dhahran, Saudi Arabia

**Keywords:** mobility limitation, patient-reported outcome measures, physical endurance, quadriceps muscle, rehabilitation, total knee arthroplasty

## Abstract

**Background:**

Functional quadriceps endurance is a key determinant of physical function after total knee arthroplasty (TKA), yet its relationship with patient-reported outcomes and the potential mediating role of mobility and balance performance remain underexplored.

**Objectives:**

To (1) examine the associations between functional quadriceps endurance and patient-reported outcomes (PROMs) after TKA, and (2) test whether dynamic balance and mobility mediate the relationship between endurance and disability.

**Methods:**

A cross-sectional study was conducted in 136 individuals (mean age 67.45 ± 6.83 years; 58 males) between 6 and 18 months post-TKA. Participants completed physical performance tests–including the 30-Second Chair Stand, Straight-Leg Raise (SLR), Timed Up and Go (TUG), Four-Square Step Test (FSST), Single-Leg Stance (SLS), and Y-Balance Test–along with PROMs (WOMAC, KOOS, PROMIS Pain Interference, LEFS). Participants were stratified into tertiles based on quadriceps endurance. Analyses included ANOVA, ANCOVA, multiple linear regression, and mediation models using Sobel tests.

**Results:**

Higher quadriceps endurance was significantly associated with better PROMs across all domains (*p* < 0.001). Regression models showed endurance independently predicted WOMAC Function (β = −0.87, *p* < 0.001), PROMIS Pain Interference (β = −0.39, *p* = 0.006), and KOOS ADL (β = 1.15, *p* < 0.001). Mediation analyses indicated significant indirect effects through TUG (β = −0.19, Sobel *p* = 0.001), FSST (β = −0.15, *p* = 0.003), and Y-Balance (β = −0.14, *p* = 0.004), supporting dynamic balance/mobility as mediators.

**Conclusion:**

Quadriceps endurance is an independent predictor of pain, function, and daily activity outcomes in TKA patients, with dynamic mobility and balance mediating its effects on disability. These findings support targeting endurance and mobility in postoperative rehabilitation.

## 1 Introduction

Total knee arthroplasty (TKA) is a widely performed surgical intervention for individuals with advanced knee osteoarthritis (OA), offering significant reductions in joint pain and improvements in function (1). Despite its clinical success, a substantial proportion of patients continue to experience limitations in mobility, balance, and activity participation even months after surgery (2). Functional recovery after TKA depends not only on surgical technique and prosthetic design but also on the restoration of neuromuscular capacity, particularly in the quadriceps muscle group (3). Rehabilitation strategies traditionally emphasize quadriceps strength as a key target, yet endurance–the ability to sustain repeated contractions or maintain submaximal force over time–plays a distinct and clinically relevant role in activities of daily living (4). Unlike strength, which reflects peak force generation, endurance is required for functional activities that involve sustained muscle use, such as walking, stair climbing, and standing for prolonged periods (5).

Several studies suggest that endurance deficits may persist longer than strength impairments and may be more predictive of functional decline in later stages of recovery (3, 6, 7). Moreover, endurance is more closely tied to postural control and repetitive task performance, both of which contribute to perceived disability (8). However, its associations with patient-reported outcomes–and the pathways through which endurance impacts recovery–remain underexplored, particularly regarding dynamic balance and mobility (8). A clearer understanding of these links is essential for optimizing rehabilitation strategies that address not only force generation but also sustained functional performance (3, 6, 7). As patients transition from postoperative recovery to reintegration into community and work environments, muscular endurance becomes increasingly critical for sustaining functional performance throughout the day (9).

Recent studies have highlighted the relationship between lower-limb muscle performance and post-TKA outcomes, with particular attention to strength, power, and motor control (6). For example, Capin et al. (10) emphasized integrating quadriceps strength and performance-based functional assessments with traditional range-of-motion and patient-reported outcomes to provide a more comprehensive evaluation of recovery after TKA. Moreover, Wei et al. (5) highlighted the central role of quadriceps strength in enhancing functional mobility, including improvements in walking ability and performance-based outcomes after TKA. However, endurance-specific assessments and their associations with patient-reported outcomes remain less frequently investigated (11). Some research, such as that by Hylkema et al. (12), noted that patients may experience persistent functional limitations in later stages of TKA recovery despite regaining strength, suggesting that unaddressed endurance deficits may contribute to perceived disability. At the same time, dynamic balance and mobility have emerged as important functional domains associated with both muscle function and patient-centered outcomes (13). Functional performance tests such as the Timed Up and Go (TUG), Four-Square Step Test (FSST), and Y-Balance Test are increasingly used to evaluate postoperative recovery, yet their potential role in mediating the effects of endurance on disability remains to be fully elucidated (14).

There is a clear need to examine the independent contribution of functional quadriceps endurance to patient-reported outcomes after TKA and to clarify the mechanistic role of dynamic balance and mobility in this relationship (15). Unlike strength, which reflects maximal force generation, muscular endurance governs the ability to perform sustained or repetitive tasks essential for prolonged daily activity. Because endurance deficits may persist even after strength recovery, assessing this domain provides unique insight into lingering functional limitations not captured by traditional strength assessments. Incorporating dynamic balance and mobility into this framework offers a more comprehensive understanding of how endurance influences disability through performance-based mechanisms relevant to real-world tasks. Although previous studies have examined associations between strength and outcomes or characterized balance impairments post-TKA, few have simultaneously modeled endurance, performance-based mobility, and disability within an integrated analytic framework (16). This gap is clinically meaningful, as it limits the precision of rehabilitation programming and the ability to identify which performance domains should be prioritized for optimal functional recovery (4). Furthermore, existing studies often conflate strength and endurance, which, although related, reflect distinct physiological constructs and functional demands (5). Addressing this gap could inform more tailored rehabilitation strategies and improve long-term outcomes for individuals undergoing TKA (5).

The primary objective of this study was to examine the association between functional quadriceps endurance and patient-reported outcomes of pain, function, and daily activity performance in individuals 6–18 months after TKA. The secondary objective was to test whether dynamic balance and mobility mediate the relationship between endurance and functional disability. It was hypothesized that greater functional quadriceps endurance would be significantly associated with better patient-reported outcomes and that this relationship would be partially mediated by mobility and balance performance metrics.

## 2 Materials and methods

### 2.1 Design and settings

This cross-sectional study was conducted between January 2023 and March 2024 at the Orthopedic Rehabilitation Clinic, Department of Physical Therapy, King Khalid University, Kingdom of Saudi Arabia. Ethical approval was obtained from the Institutional Review Board of King Khalid University (Reference No. REC# 2125-146), and written informed consent was obtained from all participants before enrollment. The study adhered fully to the ethical principles outlined in the Declaration of Helsinki.

### 2.2 Sample size calculation

*A priori* sample size calculation was conducted using G*Power 3.1 for a multiple linear regression with seven predictors, assuming a moderate effect size (f^2^ = 0.10), α = 0.05, power = 0.90, and two-tailed testing, yielding a required sample of 118 participants. To accommodate potential attrition of 15%, the target sample was increased to 136. This sample was also deemed sufficient for planned mediation analyses based on regression paths.

### 2.3 Participants

Participants were recruited via consecutive sampling from the outpatient orthopedic rehabilitation unit affiliated with the Department of Physical Therapy at King Khalid University. A total of 162 individuals were initially invited to participate. Of these, 143 met preliminary eligibility criteria and consented to screening procedures. Following exclusions due to medical contraindications or incomplete assessments (*n* = 7), the final analytic sample comprised 136 individuals, yielding a participation rate of 83.95%. Eligible individuals were referred by orthopedic consultants or identified through clinical screening records based on their postoperative follow-up visits between 6 and 18 months following unilateral TKA. The diagnosis of knee osteoarthritis leading to TKA was confirmed using the American College of Rheumatology clinical and radiographic criteria, supported by preoperative Kellgren-Lawrence grade ≥ 3 on standard weight-bearing radiographs (17).

Inclusion criteria were: (1) adults aged 50–80 years; (2) having undergone primary unilateral TKA for advanced osteoarthritis; (3) being between 6 and 18 months post-surgery; and (4) the ability to ambulate independently, with or without an assistive device. Exclusion criteria included: (1) revision TKA or bilateral procedures; (2) presence of uncontrolled medical conditions (e.g., cardiovascular or neurological disorders) that could interfere with physical testing; (3) cognitive impairment or communication barriers preventing reliable completion of questionnaires; and (4) concurrent musculoskeletal injuries or surgeries involving the contralateral limb. All prospective participants underwent an initial eligibility screening, followed by confirmation of inclusion through a medical chart review and a clinical evaluation. Baseline demographic and clinical data were collected, and trained physiotherapists administered standardized performance and questionnaire-based assessments before inclusion in the final dataset.

### 2.4 Variables

#### 2.4.1 Functional disability (WOMAC Function subscale)

The primary outcome variable in this study was self-reported functional disability, assessed using the Function subscale of the Western Ontario and McMaster Universities Osteoarthritis Index (WOMAC), version LK 3.1 (18). The WOMAC is a validated, disease-specific instrument widely used in clinical and research settings to evaluate physical function, joint stiffness, and pain in individuals with knee osteoarthritis, including those undergoing TKA (19). The Function subscale comprises 17 items assessing difficulty with activities of daily living (rising from sitting, standing, walking on flat surfaces, climbing stairs, and putting on socks), which are highly relevant to post-TKA recovery and mobility. Each item is scored on a 5-point Likert scale from 0 (“no difficulty”) to 4 (“extreme difficulty”), yielding a raw total from 0 to 68, with higher scores indicating greater functional impairment (19). The WOMAC instrument was self-administered on paper and completed under direct supervision by a trained physiotherapist to ensure accurate comprehension and minimize missing data. If participants required clarification, neutral explanations were provided without influencing responses. Scoring followed standardized guidelines and was verified by a second assessor for accuracy.

#### 2.4.2 Pain interference and ADL function (PROMIS and KOOS subscales)

Two secondary outcome variables were evaluated to capture pain-related interference and activities of daily living (ADL) function: the Patient-Reported Outcomes Measurement Information System (PROMIS) (20) Pain Interference Short Form and the Knee Injury and Osteoarthritis Outcome Score (KOOS) ADL subscale (21). The PROMIS Pain Interference instrument assesses the extent to which pain interferes with social, cognitive, emotional, physical, and recreational activities (20). It uses a 6-item short form, with each item rated on a 5-point Likert scale from “not at all” to “very much.” Responses are converted to a standardized T-score with a population mean of 50 and a standard deviation of 10; higher scores indicate greater pain interference. This norm-based scoring framework allows cross-study comparability. ADL function was measured using the KOOS ADL subscale, which consists of 17 items assessing functional limitations during daily tasks such as stair climbing, rising from sitting, and walking on flat or uneven surfaces (21). Each item is scored on a 5-point Likert scale from 0 (“no difficulty”) to 4 (“extreme difficulty”), and raw scores are transformed to a 0–100 scale, with higher values denoting better functional capacity.

In this study, both instruments were administered as part of the structured baseline assessment and completed by participants in a quiet clinical setting under investigator supervision to ensure consistency and data integrity. Their robust psychometric performance guided the selection of these instruments, responsiveness to post-operative recovery trajectories, and prior application in longitudinal outcomes research following TKA.

### 2.5 Independent variables/predictors

#### 2.5.1 Functional quadriceps endurance–30-Second Chair Stand Test (30s-CST)

The primary independent variable in this study was functional quadriceps endurance, operationalized using the 30-Second Chair Stand Test (30s-CST) (22). Although the 30s-CST reflects combined contributions of strength, endurance, and neuromuscular control, prior studies support its use as a functional proxy for lower-limb muscular endurance in clinical populations, including individuals with osteoarthritis and post-arthroplasty rehabilitation (22). This performance-based test evaluates functional lower-limb endurance by measuring the number of times a participant can rise to a full standing position from a standard-height armless chair (seat height: approximately 43–45 cm) and return to a seated position within 30 s (22). Participants were seated with their backs against the chair and their arms folded across their chests to prevent upper-limb assistance. Upon the examiner’s “go” signal, participants performed as many full stands as possible in 30 s, ensuring full hip and knee extension at the top of each repetition and a safe, controlled descent to a seated position. Incomplete or incorrectly performed repetitions (e.g., use of arms, partial stands) were not counted. A trained assessor recorded the number of valid repetitions, and participants were verbally encouraged to maintain a steady pace.

#### 2.5.2 Straight-Leg Raise (SLR) Endurance Test

Another optional indicator of quadriceps isometric endurance was the Straight-Leg Raise (SLR) Endurance Test (10), which explicitly assesses the sustained contraction capacity of the quadriceps femoris in an open-chain position. Participants lay supine with the contralateral leg flexed (knee bent, foot flat) to stabilize the pelvis. They were instructed to raise the test leg (the side of TKA) to approximately 45 degrees of hip flexion, keeping the knee fully extended, and to hold that position for as long as possible until fatigue or loss of proper form (23). Timing began when the leg reached the target height and ended if the participant (1) voluntarily terminated the hold, (2) allowed the leg to drop > 10 degrees from the target, or (3) exhibited compensatory movements such as pelvic tilting. Time to failure was recorded in seconds using a digital stopwatch. The test was terminated by the assessor if excessive fatigue, discomfort, or safety concerns were noted. This measure provides an isolated estimate of quadriceps isometric endurance and complements the more functionally integrated assessment provided by the 30s-CST.

#### 2.5.3 Covariates/confounders

To control for potential confounding, several demographic and clinical covariates were included in all adjusted analyses. These covariates included age (years), sex (male = 1, female = 0), body mass index (BMI, kg/m^2^), and time since surgery (months), all of which are known to influence recovery trajectories and physical performance after TKA. The Charlson Comorbidity Index (CCI) was also included as a measure of comorbidity burden; this validated index assigns weighted scores to chronic conditions to yield a cumulative comorbidity score predictive of functional outcomes and mortality risk (24). Additionally, participation in supervised rehabilitation (yes = 1, no = 0) was recorded, defined as attendance at structured physical therapy sessions postoperatively, which has been shown to significantly impact recovery quality and pain modulation. Including these covariates allowed for a more accurate estimation of the independent effect of functional quadriceps endurance on the study outcomes.

#### 2.5.4 Data collection instruments and procedures

All data were collected using standardized forms administered by trained physiotherapists who were calibrated before data collection. Physical performance tests were conducted in a controlled clinical environment following standardized safety procedures. Self-reported questionnaires were administered in person using paper-based forms, with clarifications provided as needed. Performance-based assessments were repeated only once to minimize fatigue effects and ensure reliability. Data were entered into a secure, password-protected electronic database and cross-checked by two independent researchers for accuracy.

### 2.6 Data analysis

Based on the study objectives, all statistical analyses were performed using IBM SPSS Statistics for Windows, Version 24.0 (IBM Corp., Armonk, NY, USA). Continuous variables were assessed for normality using the Shapiro–Wilk test and visual inspection of histograms, and parametric statistics were applied accordingly. Descriptive statistics (mean ± standard deviation) were used to summarize demographic and clinical characteristics. Independent samples *t*-tests and one-way ANOVA were conducted to compare continuous variables across sex and functional quadriceps endurance tertiles, respectively, with *post hoc* analyses (Tukey’s HSD) applied where appropriate. Chi-square tests were used to examine group differences in categorical variables. Pearson’s correlation coefficients were calculated to investigate the strength and direction of associations between functional quadriceps endurance, dynamic balance/mobility, and patient-reported outcomes. Multiple linear regression models were employed to evaluate the predictive value of functional quadriceps endurance on WOMAC Function, KOOS ADL, and PROMIS Pain Interference, adjusting for age, sex, BMI, comorbidity burden, time since surgery, and rehabilitation status. Finally, mediation analyses were performed using the causal steps approach with Sobel tests to determine whether mobility and balance measures mediated the relationship between functional quadriceps endurance and disability outcomes. Statistical significance was set at a two-tailed *p*-value of <0.05 for all analyses.

## 3 Results

As shown in Table 1, males were significantly older than females (68.79 ± 7.02 vs. 66.42 ± 6.45 years, *p* = 0.023) and had a lower body mass index (28.87 ± 3.41 vs. 30.16 ± 4.12 kg/m^2^, *p* = 0.045). A higher proportion of males were currently employed than females (44.83% vs. 28.21%, *p* = 0.031). No significant sex differences were observed for time since TKA, surgical side, implant type, hospital stay duration, comorbidity index, OA duration, participation in supervised physiotherapy, walking aid use, or fall history in the past year (*p* > 0.05 for all).

**TABLE 1:** Demographic, surgical, and clinical characteristics of the sample by sex among patients following total knee arthroplasty.

Variable	Total sample (*n* = 136)	Male (*n* = 58)	Female (*n* = 78)	*P*-value
Age (years)	67.45 ± 6.83	68.79 ± 7.02	66.42 ± 6.45	0.023
BMI (kg/m^2^)	29.61 ± 3.87	28.87 ± 3.41	30.16 ± 4.12	0.045
Education level – college or higher	72 (52.94%)	31 (53.45%)	41 (52.56%)	0.902
Currently employed	48 (35.29%)	26 (44.83%)	22 (28.21%)	0.031
Time since TKA (months)	11.46 ± 3.41	11.21 ± 3.24	11.64 ± 3.53	0.412
Side – right	78 (57.35%)	34 (58.62%)	44 (56.41%)	0.832
Implant type – PS	84 (61.76%)	37 (63.79%)	47 (60.26%)	0.781
Hospital stay (days)	4.28 ± 1.67	4.18 ± 1.61	4.35 ± 1.71	0.528
Charlson Comorbidity Index	2.13 ± 1.02	2.09 ± 1.03	2.16 ± 1.02	0.691
OA duration (years)	8.34 ± 4.95	7.96 ± 4.71	8.62 ± 5.14	0.244
Supervised physiotherapy – yes	93 (68.38%)	37 (63.79%)	56 (71.79%)	0.238
Walking aid use – yes	41 (30.15%)	17 (29.31%)	24 (30.77%)	0.913
Fall history (past 12 months) – yes	29 (21.32%)	11 (18.97%)	18 (23.08%)	0.504

TKA, total knee arthroplasty; BMI, body mass index; PS, posterior-stabilized; OA, osteoarthritis.

As presented in Table 2, patients with higher functional quadriceps endurance demonstrated significantly better performance across all dynamic balance and mobility measures (*p* < 0.001 for all). The high endurance group completed more repetitions on the 30-Second Chair Stand Test (15.98 ± 1.17) compared to moderate (13.48 ± 0.82) and low (10.98 ± 1.31) groups. Similar graded improvements were observed in SLR endurance time (65.71 ± 13.78 sec in the high group vs. 39.12 ± 10.44 sec in the low group) and in mobility tasks, including faster TUG (8.55 ± 1.06 sec vs. 11.27 ± 1.86 sec) and FSST (8.77 ± 2.65 sec vs. 12.82 ± 2.94 sec) times. Higher endurance was also associated with longer Single-Leg Stance durations on both affected (14.03 ± 6.85 sec) and non-affected sides (16.82 ± 6.45 sec), as well as higher Y-Balance composite scores (88.27% ± 5.12% vs. 77.83% ± 6.21%).

**TABLE 2:** Performance measures of functional quadriceps endurance and dynamic balance/mobility across endurance tertiles in patients following total knee arthroplasty.

Performance measure	Total sample (*n* = 136)	Low endurance (*n* = 45)	Moderate endurance (*n* = 46)	High endurance (*n* = 45)	*P*-value
30-Second Chair Stand Test (reps)	13.48 ± 3.22	10.98 ± 1.31	13.48 ± 0.82	15.98 ± 1.17	<0.001
Straight-Leg Raise (SLR) Endurance Test (sec)	52.86 ± 17.39	39.12 ± 10.44	53.76 ± 10.95	65.71 ± 13.78	<0.001
Timed Up and Go (TUG, sec)	9.82 ± 2.01	11.27 ± 1.86	9.65 ± 1.27	8.55 ± 1.06	<0.001
Four-Square Step Test (FSST, sec)	10.73 ± 3.21	12.82 ± 2.94	10.61 ± 2.31	8.77 ± 2.65	<0.001
Single-Leg Stance (affected side, sec)	10.94 ± 6.74	7.14 ± 4.19	11.62 ± 6.14	14.03 ± 6.85	<0.001
Single-Leg Stance (non-affected side, sec)	13.18 ± 7.21	9.25 ± 5.32	13.51 ± 5.91	16.82 ± 6.45	<0.001
Y-Balance Test Composite Score (% leg length)	83.61 ± 6.58	77.83 ± 6.21	84.75 ± 5.71	88.27 ± 5.12	<0.001

TUG, Timed Up and Go; FSST, Four-Square Step Test; SLR, Straight-Leg Raise.

As shown in Table 3, higher functional quadriceps endurance was significantly associated with better patient-reported outcomes across all measures, even after adjusting for age, sex, BMI, time since surgery, and comorbidities (adjusted *p* ≤ 0.017 for all). Patients in the high endurance group reported lower WOMAC Function scores (19.18 ± 8.45) than those in the moderate (25.54 ± 10.19) and low endurance (31.62 ± 11.74) groups, with the largest effect size observed in this domain (partial η^2^ = 0.182). Similar trends were evident for total WOMAC, KOOS subscales, and LEFS, with the high endurance group consistently showing reduced pain and stiffness, improved ADL and quality of life, and greater lower extremity function (e.g., KOOS ADL: 79.64 ± 13.26 vs. 64.17 ± 15.48; LEFS: 53.21 ± 9.98 vs. 41.16 ± 10.78). PROMIS Pain Interference scores decreased across tertiles (59.27–53.24), indicating less perceived impact of pain with greater endurance.

**TABLE 3:** Patient-reported outcomes by functional quadriceps endurance tertiles among patients following total knee arthroplasty.

Patient-reported outcome measure	Low endurance (*n* = 45)	Moderate endurance (*n* = 46)	High endurance (*n* = 45)	*P*-value (ANOVA)	Adjusted *P*-value (ANCOVA)	Partial η^2^
WOMAC Pain (0–20)	8.94 ± 3.11	6.85 ± 2.69	5.34 ± 2.43	<0.001	0.001	0.134
WOMAC Stiffness (0–8)	3.51 ± 1.47	2.89 ± 1.22	2.21 ± 0.98	0.004	0.017	0.067
WOMAC Function (0–68)	31.62 ± 11.74	25.54 ± 10.19	19.18 ± 8.45	<0.001	<0.001	0.182
WOMAC Total (0–96)	44.07 ± 14.23	35.28 ± 12.62	26.73 ± 10.38	<0.001	<0.001	0.165
KOOS Pain (0–100)	61.28 ± 13.56	68.74 ± 14.23	75.92 ± 12.07	<0.001	<0.001	0.148
KOOS ADL (0–100)	64.17 ± 15.48	71.55 ± 14.88	79.64 ± 13.26	<0.001	0.001	0.139
KOOS QoL (0–100)	47.82 ± 18.94	55.71 ± 17.31	64.86 ± 16.78	<0.001	<0.001	0.121
PROMIS Pain Interference (T-score)	59.27 ± 7.84	56.31 ± 6.95	53.24 ± 6.13	<0.001	0.003	0.085
LEFS (0–80)	41.16 ± 10.78	46.83 ± 11.42	53.21 ± 9.98	<0.001	<0.001	0.157

WOMAC, Western Ontario and McMaster Universities Osteoarthritis Index; KOOS, Knee Injury and Osteoarthritis Outcome Score; PROMIS, Patient-Reported Outcomes Measurement Information System; LEFS, Lower Extremity Functional Scale; ADL, activities of daily living; QoL, quality of life; ANCOVA, analysis of covariance; ANOVA, analysis of variance.

**FIGURE 1:**
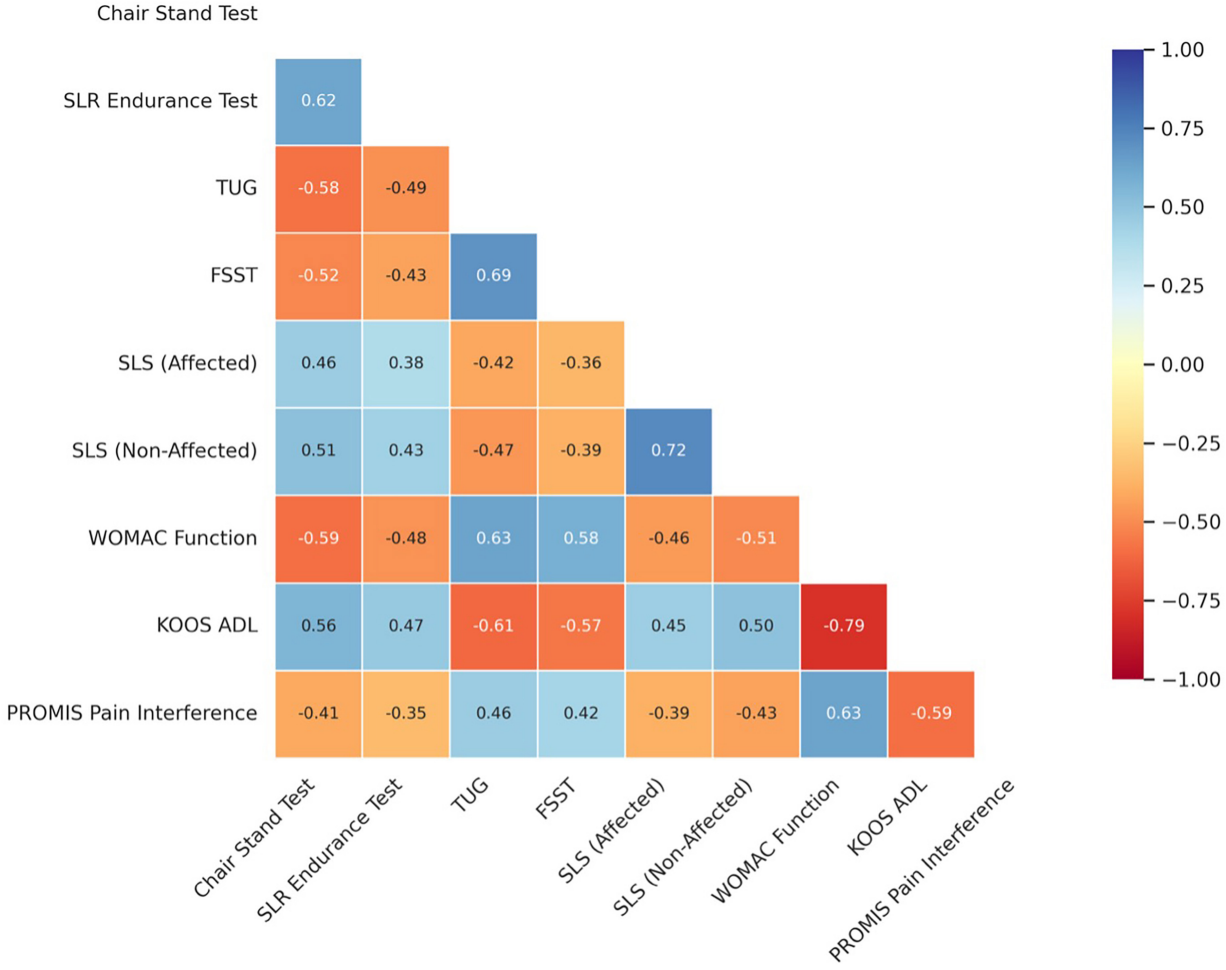
Pearson correlation matrix heatmap depicting associations between functional quadriceps endurance, balance/mobility measures, and patient-reported outcomes following total knee arthroplasty.

As presented in Figure 1, functional quadriceps endurance–as measured by the Chair Stand and SLR Endurance Tests–was strongly correlated with both physical performance and patient-reported outcomes. The Chair Stand Test showed moderate-to-strong positive correlations with SLR endurance (*r* = 0.62, *p* < 0.001), single-leg stance on both sides (*r* = 0.46–0.51, *p* < 0.001), and KOOS ADL (*r* = 0.56, *p* < 0.001), and moderate negative correlations with TUG (*r* = −0.58), FSST (*r* = −0.52), WOMAC Function (*r* = −0.59), and PROMIS Pain Interference (*r* = −0.41; all *p* < 0.001). Balance and mobility measures such as TUG and FSST were also strongly associated with PROMs, particularly with WOMAC Function (*r* = 0.63 and 0.58, respectively) and KOOS ADL (*r* = −0.61 and −0.57, respectively). Notably, higher single-leg stance durations and Y-Balance performance were consistently associated with less pain interference and better ADL function, reflecting the central role of dynamic balance and lower limb stability in mediating disability.

**FIGURE 2:**
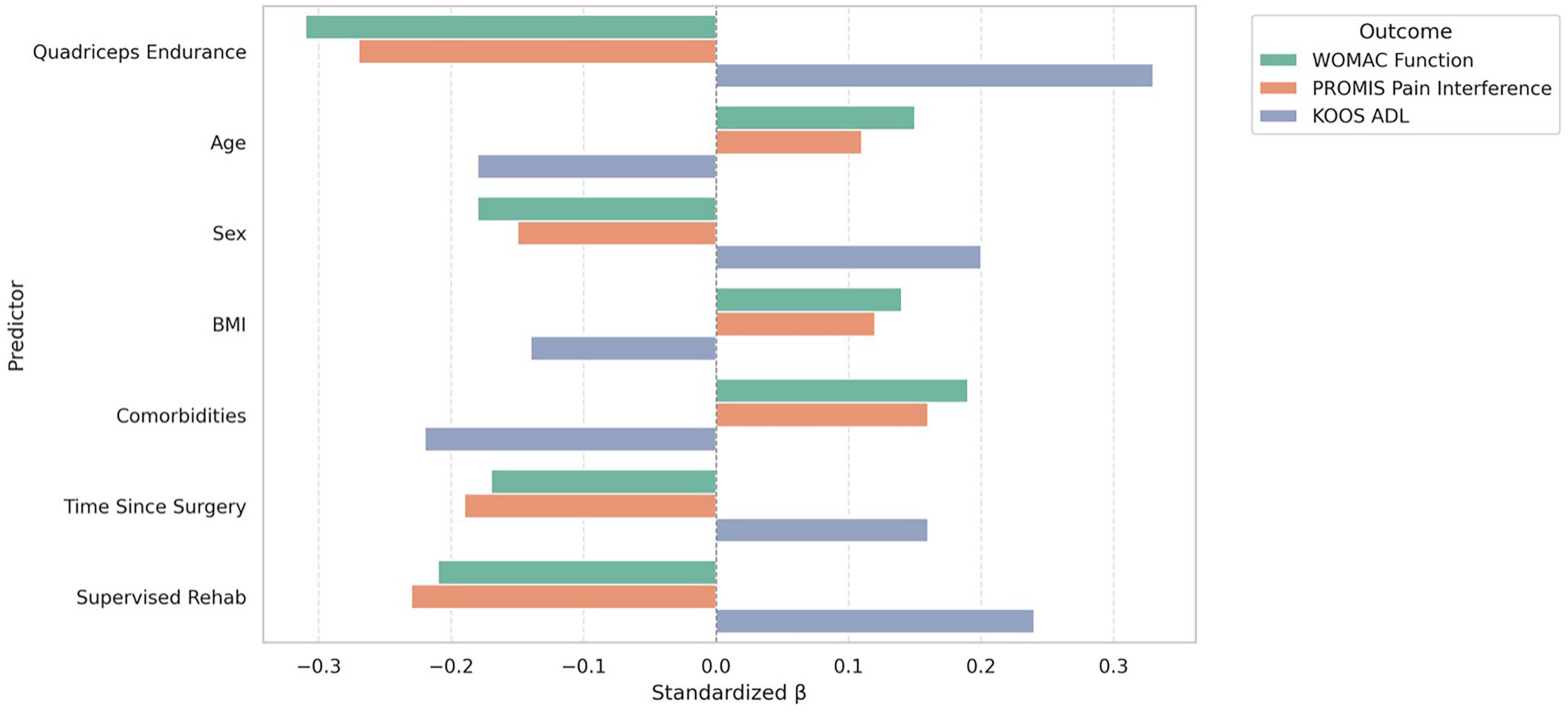
Standardized beta coefficients from multiple linear regression models predicting function, pain interference, and ADL outcomes from functional quadriceps endurance and covariates following total knee arthroplasty.

As presented in Figure 2, greater functional quadriceps endurance was a significant independent predictor of reduced disability and pain interference, as well as improved daily function, across all three regression models and functional quadriceps endurance was independently associated with reduced disability and pain interference, as well as improved daily function, across all three regression models. For WOMAC Function, higher endurance was associated with lower scores (β = −0.87, 95% CI [−1.32, −0.43], *p* < 0.001), accounting for a substantial portion of variance (Adjusted R^2^ = 0.38). Similarly, PROMIS Pain Interference decreased with increasing endurance (β = −0.39, 95% CI [−0.67, −0.11], *p* = 0.006), while KOOS ADL scores improved (β = 1.15, 95% CI [0.58, 1.72], *p* < 0.001), with the largest standardized effect seen in this domain (β = 0.33). Covariates, including comorbidity burden, age, and supervised rehabilitation, also contributed significantly to at least one model.

As shown in Table 4, all three mediation models demonstrated significant indirect effects of balance and mobility on the relationship between functional quadriceps endurance and self-reported disability. In Model 1, the effect of endurance on WOMAC Function was partially mediated by TUG performance (indirect effect β = −0.19, SE = 0.06, 95% CI [−0.31, −0.08], Sobel *p* = 0.001), with a significant attenuation of the direct effect (from β = −0.87 to β = −0.49). Similarly, Model 2 showed that FSST significantly mediated the effect of endurance on KOOS ADL (indirect effect β = −0.15, SE = 0.05, 95% CI [−0.26, −0.06], Sobel *p* = 0.003), reducing the total effect from β = 1.15 to a direct effect of β = 0.69. In Model 3, the Y-Balance Test also significantly mediated the endurance–disability link (indirect effect β = −0.14, SE = 0.05, 95% CI [−0.25, −0.04], Sobel *p* = 0.004), with the direct effect reduced to β = 0.78.

**TABLE 4:** Mediation models testing balance/mobility as mediators between functional quadriceps endurance and disability among patients following total knee arthroplasty.

Model	Path a (X → M)	Path b (M → Y | X)	Path c (X → Y total effect)	Path c′ (X → Y direct effect)	Indirect effect (a × b)
Model 1: TUG mediating between endurance and WOMAC Function	β = −0.31, SE = 0.08, *t* = −3.88, *p* < 0.001	β = 0.62, SE = 0.11, *t* = 5.64, *p* < 0.001	β = −0.87, SE = 0.23, *t* = −3.78, *p* < 0.001	β = −0.49, SE = 0.21, *t* = −2.33, *p* = 0.021	β = −0.19, SE = 0.06, 95% CI [−0.31, −0.08], Sobel *p* = 0.001
Model 2: FSST mediating between endurance and KOOS ADL	β = −0.27, SE = 0.09, *t* = −3.00, *p* = 0.003	β = 0.54, SE = 0.12, *t* = 4.50, *p* < 0.001	β = 1.15, SE = 0.29, *t* = 3.97, *p* < 0.001	β = 0.69, SE = 0.25, *t* = 2.76, *p* = 0.007	β = −0.15, SE = 0.05, 95% CI [−0.26, −0.06], Sobel *p* = 0.003
Model 3: Y-Balance mediating between endurance and KOOS ADL	β = 0.33, SE = 0.10, *t* = 3.30, *p* = 0.001	β = −0.41, SE = 0.13, *t* = −3.15, *p* = 0.002	β = 1.15, SE = 0.29, *t* = 3.97, *p* < 0.001	β = 0.78, SE = 0.26, *t* = 3.00, *p* = 0.003	β = −0.14, SE = 0.05, 95% CI [−0.25, −0.04], Sobel *p* = 0.004

TUG, Timed Up and Go; FSST, Four-Square Step Test; WOMAC, Western Ontario and McMaster Universities Osteoarthritis Index; KOOS, Knee Injury and Osteoarthritis Outcome Score; ADL, activities of daily living; SE, standard error; CI, confidence interval.

## 4 Discussion

This study aimed to characterize the relationship between functional quadriceps endurance and functional outcomes in individuals following TKA, with a particular focus on its association with patient-reported disability and the mediating role of dynamic balance and mobility. The findings demonstrated that greater functional quadriceps endurance was consistently associated with better physical performance and more favorable self-reported outcomes across multiple domains, including pain, functional limitations, and participation in daily activities. Regression analyses confirmed that endurance independently predicted lower disability and pain interference and higher ADL function, even after adjusting for relevant clinical covariates. Furthermore, mediation models indicated that balance and mobility measures, such as TUG, FSST, and Y-Balance, significantly mediated the relationship between endurance and disability, underscoring their mechanistic role in the pathway linking muscular performance to functional outcomes after TKA.

The results demonstrated that higher functional quadriceps endurance was independently associated with lower levels of self-reported pain and disability and better function in daily activities. These associations remained significant even after adjusting for demographic and clinical covariates, including age, sex, BMI, comorbidity burden, time since surgery, and participation in supervised rehabilitation (25). These findings align with previous literature emphasizing the critical role of lower limb muscular capacity in influencing patient-perceived recovery after TKA (26). For instance, Kim et al. (27) reported that quadriceps strength accounted for substantial variance in physical function outcomes early after TKA. Similarly, Dutta et al. (28) highlighted the importance of muscular endurance in sustaining joint stability and reducing compensatory movement patterns, which may contribute to improved function and reduced symptom burden. Moreover, findings by Yoshida et al. (29) suggested that endurance-specific deficits in the quadriceps may be more strongly associated with long-term functional limitations than peak strength alone. These studies support the present findings by illustrating that muscular endurance is a distinct, clinically relevant component of lower extremity performance that directly influences patients’ daily functioning and perception of disability (29, 30).

All three mediation models–featuring the TUG, FSST, and Y-Balance tests–indicated statistically significant indirect effects, suggesting that dynamic balance and mobility play a key mechanistic role in translating muscular endurance into functional outcomes (5, 6). These findings are consistent with prior studies that have established mobility-related tasks as sensitive indicators of post-TKA functional status. For example, Vertesich et al. (31) demonstrated that TUG performance is closely linked to both lower limb strength and patient-reported activity limitations. Likewise, Lee et al. (13) found that impaired balance and motor control contribute significantly to perceived functional deficits in individuals with knee osteoarthritis and post-TKA recovery. Additionally, de Maio Nascimento et al. (32) identified dynamic balance as a mediating factor in the relationship between muscle capacity and self-reported disability among older adults with musculoskeletal impairments. These prior findings lend empirical support to the current results, reinforcing the concept that improvements in functional quadriceps endurance may enhance physical function not only directly but also indirectly through their positive impact on postural stability and mobility efficiency (33, 34). These results collectively support a clinically meaningful correlation: individuals with higher functional quadriceps endurance experience significantly better functional outcomes after TKA, as measured by both performance-based tests and patient-reported assessments, with dynamic balance and mobility playing key explanatory roles.

### 4.1 Clinical significance

The findings of this study underscore the clinical relevance of assessing and targeting functional quadriceps endurance in the rehabilitation of individuals following TKA. Functional quadriceps endurance was independently associated with key patient-reported outcomes, including pain, functional limitation, and participation in daily activities, and its impact was partially mediated by dynamic balance and mobility performance. These results suggest that interventions specifically designed to improve muscular endurance–not just strength–may offer functional benefits that translate to meaningful improvements in perceived recovery. The strong mediating role of mobility-related performance further highlights the importance of incorporating dynamic balance and agility training into postoperative rehabilitation protocols to optimize patient-centered outcomes after TKA.

### 4.2 Limitations and areas for future research

Several limitations should be considered when interpreting the results of this study. First, the cross-sectional design precludes causal inferences regarding the relationships between functional quadriceps endurance, mobility, and self-reported outcomes. Second, although a comprehensive set of covariates was included in the regression and mediation models, unmeasured factors, such as psychological status, pain coping strategies, or variations in surgical technique, may have influenced the observed associations. Third, endurance was operationalized using functional field tests, particularly the 30s-CST, which reflects global lower-extremity function and may not isolate functional quadriceps endurance. While the test is clinically relevant, it encompasses multiple physiological domains, including strength and balance, limiting its specificity. Future longitudinal studies are warranted to examine the temporal sequencing of these associations and to evaluate the effectiveness of targeted endurance and balance interventions on long-term functional recovery after TKA.

## 5 Conclusion

This study demonstrated that greater functional quadriceps endurance is independently associated with better self-reported function, lower pain interference, and enhanced activities of daily living in individuals following total knee arthroplasty. Furthermore, the relationship between endurance and disability was partially mediated by mobility and dynamic balance performance, indicating that these factors play a mechanistic role in the pathway linking muscular endurance to functional outcomes. These findings highlight functional quadriceps endurance and mobility performance as clinically meaningful therapeutic targets in post-TKA rehabilitation.

## Data Availability

The datasets presented in this study can be found in online repositories. The names of the repository/repositories and accession number(s) can be found in the article/supplementary material.

## References

[1] Zhang Y Liu H. Safety of total knee arthroplasty in the treatment of knee osteoarthritis and its effect on postoperative pain and quality of life of patients. *Contrast Media Mol Imaging*. (2021) 2021:6951578. 10.1155/2021/6951578 35024014 PMC8716239

[2] Taylor C Murray C Stanton T. Patient perspectives of pain and function after knee replacement: a systematic review and meta-synthesis of qualitative studies. *Pain Rep*. (2022) 7:e1006. 10.1097/PR9.0000000000001006 35558092 PMC9088230

[3] Paravlic A Meulenberg C Drole K. The time course of quadriceps strength recovery after total knee arthroplasty is influenced by body mass index, sex, and age of patients: systematic review and meta-analysis. *Front Med*. (2022) 9:865412. 10.3389/fmed.2022.865412 35692543 PMC9174520

[4] Konnyu K Thoma L Cao W Aaron R Panagiotou O Bhuma M Rehabilitation for total knee arthroplasty: a systematic review. *Am J Phys Med Rehabil*. (2023) 102:19–33. 10.1097/PHM.0000000000002008 35302953 PMC9464796

[5] Wei G Shang Z Li Y Wu Y Zhang L. Effects of lower-limb active resistance exercise on mobility, physical function, knee strength and pain intensity in patients with total knee arthroplasty: a systematic review and meta-analysis. *BMC Musculoskelet Disord*. (2024) 25:730. 10.1186/s12891-024-07845-9 39267026 PMC11395693

[6] Singla R Niederer D Franz A Happ K Zilkens C Wahl P The course of knee extensor strength after total knee arthroplasty: a systematic review with meta-analysis and -regression. *Arch Orthop Trauma Surg*. (2023) 143:5303–22. 10.1007/s00402-022-04750-5 36637491 PMC10374784

[7] Edwards R Campbell C Schreiber K Meints S Lazaridou A Martel M Multimodal prediction of pain and functional outcomes 6 months following total knee replacement: a prospective cohort study. *BMC Musculoskelet Disord*. (2022) 23:302. 10.1186/s12891-022-05239-3 35351066 PMC8966339

[8] An J Son Y Lee B. Effect of combined kinematic chain exercise on physical function, balance ability, and gait in patients with total knee arthroplasty: a single-blind randomized controlled trial. *Int J Environ Res Public Health*. (2023) 20:3524. 10.3390/ijerph20043524 36834218 PMC9961064

[9] Sun J Xu Y Zhu J Zhu B Gao W. Efficacy and safety of continuous nursing in improving functional recovery after total hip or knee arthroplasty in older adults: a systematic review. *Int J Nurs Sci*. (2024) 11:286–94. 10.1016/j.ijnss.2024.03.013 38707686 PMC11064567

[10] Capin J Bade M Jennings J Snyder-Mackler L Stevens-Lapsley J. Total knee arthroplasty assessments should include strength and performance-based functional tests to complement range-of-motion and patient-reported outcome measures. *Phys Ther*. (2022) 102:zac033. 10.1093/ptj/pzac033 35358318 PMC9393064

[11] Hashizaki T Nishimura Y Ogawa T Ohno C Kouda K Umemoto Y Effectiveness of a 3-week rehabilitation program combining muscle strengthening and endurance exercises prior to total knee arthroplasty: a non-randomized controlled trial. *J Clin Med*. (2023) 12:1523. 10.3390/jcm12041523 36836057 PMC9967873

[12] Hylkema T Brouwer S Stewart R van Beveren J Rijk P Brouwer R Two-year recovery courses of physical and mental impairments, activity limitations, and participation restrictions after total knee arthroplasty among working-age patients. *Disabil Rehabil*. (2022) 44:291–300. 10.1080/09638288.2020.1766583 32441539

[13] Lee H An J Lee B. The effect of progressive dynamic balance training on physical function, the ability to balance and quality of life among elderly women who underwent a total knee arthroplasty: a double-blind randomized control trial. *Int J Environ Res Public Health*. (2021) 18:2513. 10.3390/ijerph18052513 33802559 PMC7967306

[14] Schwartz I Kandel L Sajina A Litinezki D Herman A Mattan Y. Balance is an important predictive factor for quality of life and function after primary total knee replacement. *J Bone Joint Surg Br*. (2012) 94:782–6. 10.1302/0301-620X.94B6.27874 22628592

[15] Gauchard G Vançon G Meyer P Mainard D Perrin P. On the role of knee joint in balance control and postural strategies: effects of total knee replacement in elderly subjects with knee osteoarthritis. *Gait Posture*. (2010) 32:155–60. 10.1016/j.gaitpost.2010.04.002 20451390

[16] Prochnicki B. *Functional Outcomes Following Total Knee Arthroplasty Utilizing Lifestyle Risk Factors and Comorbidities on Performance-Based Tests.* Thunder Bay: Lekehead University (2024).

[17] Van Manen M Nace J Mont M. Management of primary knee osteoarthritis and indications for total knee arthroplasty for general practitioners. *J Am Osteopath Assoc*. (2012) 112:709–15. 10.7556/jaoa.2012.112.11.709 23139341

[18] McConnell S Kolopack P Davis A. The Western Ontario and McMaster universities Osteoarthritis index (WOMAC): a review of its utility and measurement properties. *Arthritis Rheum*. (2001) 45:453–61. 10.1002/1529-0131(200110)45:5<453::aid-art365>3.0.co;2-w 11642645

[19] Ab Rahman S Narhari P Sharifudin M Shokri A. Western Ontario and McMaster universities (WOMAC) Osteoarthritis index as an assessment tool to indicate total knee arthroplasty in patients with primary knee osteoarthritis. *IIUM Med J Malaysia.* (2020) 19:47–53. 10.31436/imjm.v19i3.1664

[20] Czerwonka N Gupta P Desai S Hickernell T Neuwirth A Trofa D. Patient-reported outcomes measurement information system instruments in knee arthroplasty patients: a systematic review of the literature. *Knee Surg Relat Res*. (2023) 35:27. 10.1186/s43019-023-00201-6 38041197 PMC10690965

[21] Nishimoto J Tanaka S Inoue Y Tanaka R. Minimal clinically important differences in short-term postoperative Knee injury and Osteoarthritis outcome score (KOOS) after total knee arthroplasty: a prospective cohort study. *J Orthopaedics Trauma Rehabil.* (2024) 31:15–20. 10.1177/22104917231181644

[22] Simpson E Mohammadi S Miller W Yu N Watson W Westby M. Reliability of conducting the 30-second chair stand test virtually among individuals with osteoarthritis. *Arch Rheumatol Arthritis Res.* (2022) 2:1–4. 10.33552/ARAR.2022.02.000542

[23] Suh J Liow M Pua Y Chew E Chia S Lo N Early postoperative straight leg raise is associated with shorter length of stay after unilateral total knee arthroplasty. *J Orthop Surg*. (2021) 29:23094990211002294. 10.1177/23094990211002294 33779408

[24] Lee K Chang W Tsai S Chen C Wu P Chen W. The impact of Charlson Comorbidity Index on surgical complications and reoperations following simultaneous bilateral total knee arthroplasty. *Sci Rep*. (2023) 13:6155. 10.1038/s41598-023-33196-x 37061607 PMC10105729

[25] Choi J Kim B Kim S Nam K Lee S Kim W Physical performance correlates with self-reported physical function and quality of life in patients at 3 months after total knee arthroplasty. *Ann Geriatr Med Res*. (2020) 24:99–106. 10.4235/agmr.20.0018 32743330 PMC7370793

[26] Crosbie J Naylor J Harmer A Russell T. Predictors of functional ambulation and patient perception following total knee replacement and short-term rehabilitation. *Disabil Rehabil*. (2010) 32:1088–98. 10.3109/09638280903381014 19860602

[27] Kim Y Choi S Park J Yang H Lee W Kim S Low phase angle indicates poor muscle strength and physical performance in patients with knee osteoarthritis awaiting total knee arthroplasty. *Sci Rep*. (2025) 15:27511. 10.1038/s41598-025-13065-5 40721851 PMC12304258

[28] Dutta S Ambade R Wankhade D Agrawal P. Rehabilitation techniques before and after total knee arthroplasty for a better quality of life. *Cureus*. (2024) 16:e54877. 10.7759/cureus.54877 38533163 PMC10965116

[29] Yoshida Y Mizner R Snyder-Mackler L. Association between long-term quadriceps weakness and early walking muscle co-contraction after total knee arthroplasty. *Knee*. (2013) 20:426–31. 10.1016/j.knee.2012.12.008 23352711 PMC3692574

[30] Morelli I Maffulli N Brambilla L Agnoletto M Peretti G Mangiavini L. Quadriceps muscle group function and after total knee arthroplasty-asystematic narrative update. *Br Med Bull*. (2021) 137:51–69. 10.1093/bmb/ldaa041 33517365

[31] Vertesich K Staats K Schneider E Willegger M Windhager R Böhler C. Balance and mobility in comparison to patient-reported outcomes-a longitudinal evaluation after total hip and knee arthroplasty. *J Clin Med*. (2025) 14:4135. 10.3390/jcm14124135 40565883 PMC12193784

[32] de Maio Nascimento M Gouveia B Gouveia ÉR Campos P Marques A Ihle A. Muscle strength and balance as mediators in the association between physical activity and health-related quality of life in community-dwelling older adults. *J Clin Med*. (2022) 11:4857. 10.3390/jcm11164857 36013095 PMC9409764

[33] Yurttaş A Aracı A Akkoyun Sert Ö Ismayılov T. Investigation of knee joint position sensation balance reaction time and function in individuals with knee osteoarthritis and unilateral total knee arthroplasty. *J Back Musculoskelet Rehabil*. (2025): 10.1177/10538127251387392 Online ahead of print. 41143951

[34] Taniguchi M Sawano S Maegawa S Ikezoe T Ichihashi N. Physical activity mediates the relationship between gait function and fall incidence after total knee arthroplasty. *J Knee Surg*. (2021) 34:1205–11. 10.1055/s-0040-1702165 32131104

